# Tailoring Dynamically At-Risk Airway Management in an Impacted Safety Pin: A Near-Miss Case Report

**DOI:** 10.7759/cureus.117967

**Published:** 2026-10-15

**Authors:** Pravin K, Bhabana Talukdar, Aakriti Chandra, Habib Md R Karim

**Affiliations:** 1 Anesthesiology, Critical Care, and Pain Medicine, All India Institute of Medical Sciences Guwahati, Assam, IND; 2 Otolaryngology - Head and Neck Surgery, All India Institute of Medical Sciences Guwahati, Assam, IND

**Keywords:** awake video laryngoscopy, identify at-risk patients, near miss event, open safety-pin, pharyngeal foreign body

## Abstract

Sharp foreign bodies lodged in the pharynx represent an airway emergency that is more than a single fixed anatomical problem. A 13-year-old girl presented after accidental insertion of an open safety pin into the nasopharyngeal region, with the pin having a descending trajectory towards the laryngopharynx. On examination, there was mucosal swelling with minimal oozing. Potential complications included loss of spontaneous airway tone, with a risk of posterior migration of the pin; conversion of oozing to active bleeding from accidental foreign body manipulation during airway management; and aspiration related to bleeding. Further, the patient was not fasted, and shared airway management and anesthesia maintenance with the surgical team also added to the challenges. The plan, therefore, focused on securing the airway while preserving spontaneous breathing under sedation, using video laryngoscopy (VL). Awake fibreoptic intubation (AFOBI) was considered but was thought to be technically difficult due to a sharp foreign body in the nasopharynx, and the possibility of conversion into active bleeding if it were manipulated. The airway was secured on second attempt using a C-MAC D-blade, and the foreign body was subsequently retrieved surgically. A brief episode of mild desaturation was noted by the time the trachea was intubated. This case highlights the compound and dynamic airway risks associated with sharp foreign-body impaction, which should be managed as a near-miss requiring structured, awake, shared-airway planning. In our case, VL with preserved spontaneous breathing effort seemed a rational first-line technique given the technical difficulties and bleeding risk that could make fibreoptic visualization unreliable, and it offered the potential advantages of tolerance, less force, and a higher likelihood of no-touch to the palate during laryngoscopy compared with conventional direct laryngoscopy.

## Introduction

Insertion and impaction of sharp metallic foreign bodies, such as open safety pins, are recognized emergencies. Although it most commonly occurs in children under five, it can also occur in older children and adolescents [1]. When an object lodges in the nasopharynx, hypopharynx, or laryngeal inlet, the anesthetic and surgical teams face an anatomically compromised airway, a risk of further injury, and a shared airway between two operators with competing requirements.

Airway management in such cases can be highly challenging, particularly when the foreign body is located near the glottis or when obstruction and bleeding are present. Although direct laryngoscopy is often feasible, some cases may require advanced techniques, including cricothyrotomy [2]. Active bleeding associated with sharp foreign bodies, together with the possibility of dislodgement during airway instrumentation, also influences the airway management plan. Although awake fibreoptic intubation (AFOBI) is conventionally regarded as a gold-standard technique for anticipated difficult airway management, its use can be particularly challenging in children when the foreign body is located in the nasopharynx. Collectively, these factors can rapidly lead to airway management failure and catastrophic consequences. Most published accounts of pin-in-airway cases focus on the surgical retrieval technique [3,4]. Few frame anesthetic decision-making as a perioperative near-miss: a situation in which multiple independent failure pathways converge, and any one of them could turn a controlled plan into a cannot-intubate, cannot-oxygenate emergency within seconds [5,6]. With the parents' informed consent, we present this case to document that reasoning, as a well-managed near-miss produces no major adverse outcome and therefore may rarely generate an incident report through routine channels.

## Case presentation

A 13-year-old girl of average build for her height, weighing approximately 40 kg, presented with a history of accidental insertion of an open safety pin, suggestive of foreign-body impaction in the nasopharynx. She reported difficulty and painful swallowing, with blood-tinged saliva. On presentation, she was stable, with a blood pressure of 118/74 mmHg and a heart rate of 94 beats per minute, and was maintaining her own airway with room air saturation of 99%. She had no significant medical, psychological, or surgical history and no known allergies in herself or her family. She was a little worried but co-operative, and oral examination showed ongoing minor oropharyngeal bleeding. A lateral soft-tissue neck radiograph showed an open safety pin, with the long axis positioned so the hinge and one limb were in the nasopharyngeal region; no free air or gross evidence of soft tissue edema and deeper tissue penetration was evident. Cervical spine alignment was preserved (Figure 1).

**Figure 1:**
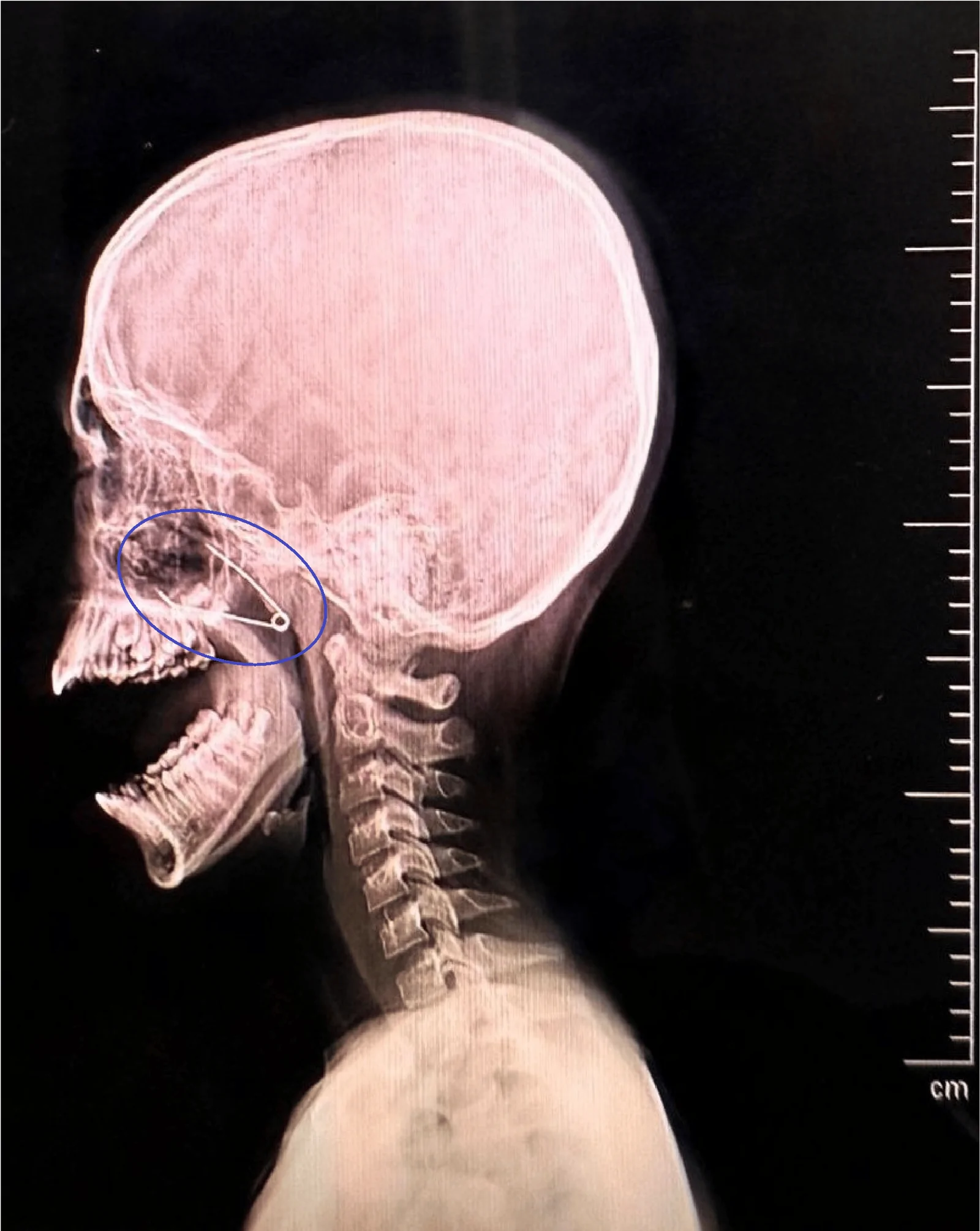
Lateral view x-ray with mouth open showing a radio-opaque foreign body consistent with a safety pin; the sharp pointed end is in the nasopharynx and the hinge projecting in nasopharyngeal-oropharyngeal junction

Airway assessment deemed the patient high risk, not because of a static anatomical predictor but because of dynamic threats. The surgical team agreed to use a shared-airway protocol, and a rescue emergency front-of-neck access plan was also ready. After deliberation, the anesthesiologist chose video laryngoscopy (VL) to secure the airway. The airway, especially the oropharyngeal inlet, faucial pillars, base of the tongue, and posterior pharynx, was topicalized with 10% lignocaine spray (two sprays). A muscle-relaxant-free induction technique was planned, with the patient breathing spontaneously to preserve airway reflexes and tone. Intravenous fentanyl 30 µg and ketamine 40 mg were administered, with minimally assisted positive-pressure ventilation by bag and mask using 100% oxygen. The patient's lower jaw was gently assisted open while a command to open the mouth was given, and a VL blade (C-Mac 3 D-blade from KARL STORZ SE & Co. KG, Germany) was inserted while taking care not to touch the hard and soft palate. Under direct vision, blood and secretions were suctioned; the anterior larynx was noted with Cormack-Lehane grade IIb. The airway was successfully secured on the second attempt by the same resident while changing the stylet angulation and using a 5.5 cuffed flexometallic tube. Tube placement was confirmed with end-tidal carbon dioxide and auscultation. It took nearly five minutes from the start of sedation to tracheal intubation confirmation. By this time, the patient had a brief episode of desaturation (saturation fell to 94%), which improved after controlled ventilation with 100% oxygen. Subsequently, endotracheal suctioning and hydrocortisone 100 mg were also administered. After securing the airway, a 30-mg bolus of propofol and cis-atracurium 4.5 mg were administered, and general anesthesia was maintained with sevoflurane titrated to an age-adjusted minimum alveolar concentration of 1 +/- 0.1. Once the patient was stabilized, the surgical team retrieved the pin using standard endoscopic instruments.

Removal of the foreign body caused additional mucosal injury and bleeding; hemostasis was achieved after pin removal using bipolar cautery. The patient was extubated once her airway reflexes had fully returned. No further complications or events were noted. Postprocedure recovery was normal, and the patient was discharged from the postanesthesia care unit to the ward after an hour. No further steroid therapy was advised by the anesthesia team.

## Discussion

We present this case as a near-miss rather than a difficult airway, anticipated or otherwise. The difficulty was compound and dynamic, with several factors that could convert a stable situation into an emergency during induction or instrumentation. This included loss of spontaneous airway tone following administration of a sedative or induction agent and positive-pressure bag-mask ventilation; aspiration risk associated with active oozing with a potential for conversion into active bleeding, a non-fasted stomach, and blood pooling in the hypopharynx above the cords; possible visualization failure and local mucosal edema or distortion; and, last but not least, the shared airway with the surgical team.

While airway guidelines describe management pathways for anticipated and unanticipated difficulties and emphasize shifting from rescue-based strategies to first-attempt success, the situation's dynamic nature can make applying a predefined roadmap difficult or impractical at certain points [5,7,8]. In our case, the patient had an open, sharp metallic foreign body with minor ongoing oozing and edema. This made placement of a nasopharyngeal airway impractical; even inserting an oropharyngeal or supraglottic airway could have disturbed the foreign body in situ and caused further bleeding. Nevertheless, even during conventional orotracheal intubation, the laryngoscope blade may touch or press the soft palate and push cephalad, causing active bleeding and jeopardizing the airway. On the other hand, oral AFOBI requires placing a bite-block and railroading the endotracheal tube, which can still cause displacement or injury from the sharp foreign body. Further, fully awake procedures often require high patient co-operation; mild to moderate sedation is frequently used. Thus, we planned VL under moderate sedation, with airway topicalization, while preserving spontaneous breathing effort, assisting jaw opening, and keeping the target area in view without touching the soft palate. These considerations required agreement on a plan tailored to mitigate the anticipated risks. Maintaining spontaneous breathing efforts with agents that preserve protective airway reflexes to some extent and maintain muscle tone, choosing VL over AFOBI, and having a pre-briefed shared-airway plan covering suction-first management, limits of extension, and rescue criteria proved effective in our case. Although AFOBI has traditionally been considered the gold standard for anticipated difficult airway management, limitations in visualization due to blood or secretions, a non-trivial failure rate even among experienced practitioners, and the potential for serious complications, including bleeding, show that this technique is not inherently risk-free [5,9,10]. Furthermore, meta-analyses have shown awake VL to be significantly faster than AFOBI, with comparable success and complication rates [11]. It is also well tolerated and allows active suctioning of the surgical field under direct vision, which was most relevant in our case in the event of bleeding.

Our patient had mild desaturation by the time the trachea was intubated, possibly because the time from sedation to tracheal intubation was five minutes; the patient was young, likely had high demand, and had depressed respiration with sedation. Although assisted, the assistance was not full, but limited to maintaining airway patency and providing slight support during inspiratory effort.

We do not claim that the technique employed is the only possible plan; conventional laryngoscopy with standard induction could also work. We want to emphasize the rationale for not following the conventional path in such a dynamically at-risk airway. We also do not claim the drugs used were the absolute best; spontaneous breathing during anesthesia and sedation can be maintained with agents such as dexmedetomidine, sevoflurane, remifentanil, or helium, and these approaches have been successfully used in airway foreign-body removal [12,13].

Our case highlights that managing a dynamically at-risk difficult airway requires an intuitively "safe" choice of approach, team, and technique to avoid turning a controlled situation into an airway emergency. While some may debate the best plan, the main lesson is that the overall strategy should be appropriate and context-specific, even if it is not universally applicable. This is precisely what near-miss reporting is designed to capture, because a properly managed near-miss causes no harm and might otherwise be overlooked.

## Conclusions

An impacted sharp foreign body, such as an open safety pin, in the oro-nasopharynx and should be viewed as a compound, dynamic airway threat and an at-risk difficult airway, even though anatomical bedside airway assessment does not categorize it as an anticipated difficult airway. In the setting of active oozing with potential to convert to active bleeding, VL with preserved spontaneous breathing effort under mild-to-moderate sedation and airway topicalization may be a rational, evidence-based approach, with the advantages of permitting active suction under vision. The uneventful outcome, except for a brief clinically or practically insignificant desaturation in this case, depended on structured, pre-briefed, multidisciplinary planning, including explicit shared-airway negotiation with the surgical team and a tailored induction and laryngoscopy technique. Nevertheless, this single case does not claim that the technique used or drugs used were the only options or best options. 
